# Evidence for *Hox*-specified positional identities in adult vasculature

**DOI:** 10.1186/1471-213X-8-93

**Published:** 2008-09-30

**Authors:** Nathanael D Pruett, Richard P Visconti, Donna F Jacobs, Dimitri Scholz, Tim McQuinn, John P Sundberg, Alexander Awgulewitsch

**Affiliations:** 1 Department of Medicine, Medical University of South Carolina, 96 Jonathan Lucas Street, Charleston, SC 29425, USA; 2 Department of Cell Biology and Anatomy, Medical University of South Carolina, 173 Ashley Avenue, Charleston, SC 29425, USA; 3 Department of Pediatrics, Division of Pediatric Cardiology, Medical University of South Carolina, 171 Ashley Avenue, Charleston, SC 29425, USA; 4 The Jackson Laboratory, 600 Main Street, Bar Harbor, ME 04609, USA; 5 Conway Institute of Biomolecular and Biomedical Research, University College Dublin, Dublin 4, Ireland

## Abstract

**Background:**

The concept of specifying positional information in the adult cardiovascular system is largely unexplored. While the *Hox *transcriptional regulators have to be viewed as excellent candidates for assuming such a role, little is known about their presumptive cardiovascular control functions and *in vivo *expression patterns.

**Results:**

We demonstrate that conventional reporter gene analysis in transgenic mice is a useful approach for defining highly complex *Hox *expression patterns in the adult vascular network as exemplified by our *lacZ *reporter gene models for *Hoxa3 *and *Hoxc11*. These mice revealed expression in subsets of vascular smooth muscle cells (VSMCs) and endothelial cells (ECs) located in distinct regions of the vasculature that roughly correspond to the embryonic expression domains of the two genes. These reporter gene patterns were validated as authentic indicators of endogenous gene expression by immunolabeling and PCR analysis. Furthermore, we show that persistent reporter gene expression in cultured cells derived from vessel explants facilitates *in vitro *characterization of phenotypic properties as exemplified by the differential response of *Hoxc11-lacZ*-positive *versus*-negative cells in migration assays and to serum.

**Conclusion:**

The data support a conceptual model of *Hox-*specified positional identities in adult blood vessels, which is of likely relevance for understanding the mechanisms underlying regional physiological diversities in the cardiovascular system. The data also demonstrate that conventional *Hox *reporter gene mice are useful tools for visualizing complex *Hox *expression patterns in the vascular network that might be unattainable otherwise. Finally, these mice are a resource for the isolation and phenotypic characterization of specific subpopulations of vascular cells marked by distinct *Hox *expression profiles.

## Background

The *Hox *transcriptional regulators are known to play a critical role in establishing positional identities during embryonic patterning [1], whereas in postnatal and adult tissues, their functions are largely subject to speculation [2]. This also pertains to the adult cardiovascular system, although *Hox *genes are considered prime candidates for determining phenotypic characteristics of vascular smooth muscle cells (VSMCs) and endothelial cells (ECs) during vasculogenesis and vascular remodeling both under normal (e.g. wound healing, menstrual cycle) and pathologic conditions (e.g. cancer-related angiogenesis, atherosclerosis) [3]. A prerequisite for defining these roles and underlying molecular mechanisms is information about *Hox *expression patterns in adult vasculature *in vivo*, which is currently scarcely available.

Considering the documented expression of various members of this gene family in ECs and VSMCs [3], the near-void of data concerning phenotypic changes in the cardiovascular system of the numerous *Hox *gene-targeted and transgenic mice is surprising. A notable exception is the *Hoxa3 *gene-targeted mice, which exhibit a range of cardiovascular defects [4-6], although a definition of the molecular mechanisms underlying these defects was essentially precluded due to lack of information about the cardiovascular *Hoxa3 *expression pattern, both in the embryo and the adult. Perhaps of equal relevance is that in the fruit fly *Drosophila melanogaster *the *Hox *gene *abdA *is required for specifying cell identity in a posterior section of the *Drosophila *dorsal vessel, which is functionally equivalent to the vertebrate heart [7].

Since these seminal observations with *Hoxa3 *and *abdA *in mice and flies, respectively, a series of studies has shown differential expression of *Hox *genes in ECs of different origin with respect to species and vessel type. For example, 8 of the 10 human *HOXB *genes were found expressed in cultured umbilical vein ECs and this expression could be modulated by vascular signaling molecules including tissue plasminogen activator (TPA), and vascular endothelial growth factor (VEGF) [8]. Most of the studies involving endothelial *Hox *expression suggest a role in the regulation of angiogenesis, i.e. the formation of new blood vessels and microvasculature associated either with normal developmental and physiological processes such as mammary gland development and wound healing or with pathological conditions such as tumorigenesis [8-16].

Evidence for *Hox *expression in VSMCs was initially provided by showing *Hoxa2 *expression in VSMCs of embryonic vessels leading from the heart, in embryonic cardiomyocytes, and in adult aortic VSMCs [17]; this was facilitated by the isolation of a *Hoxa2*-specific cDNA from a rat aorta-derived cDNA library. Additional VSMC-specific *Hox *cDNAs *(HOXA5, HOXA11, HOXB1, HOXB7*, and *HOXC9) *were isolated from fetal and adult human VSMC cDNA libraries by using degenerate primers corresponding to a highly conserved subregion of the homeobox for screening [18]. Although providing more direct evidence for *in vivo *expression of any of the corresponding genes in fetal or adult aorta remained elusive in that study, the potential relevance of these data was underscored in a separate study showing scattered expression of *HOXB7 *in media and neointima adjacent to calcification in human atherosclerotic plaques [19]. To assess whether this might reflect a role in directing immature cells towards either osteoblastic or VSMC differentiation, *HOXB7 *was overexpressed in multipotent C3H10T1/2 cells that are capable of differentiating into VSMCs, as well as osteogenic and chondrogenic lineages. The results showed 3.5-fold increase in proliferation and induction of VSMC-like morphology, thus suggesting a role for *HOXB7 *in the expansion of immature cell populations or dedifferentiation of mature cells [19]. Of relevance in this context is the apparent VSMC phenotype-dependent expression of *HOXB7 *and *HOXC9 *as evidenced by preferential expression in cultured fetal versus adult VSMCs, a result that might indicate a role for *Hox *genes in the control of VSMC-diversity [18].

Although the activity patterns in different cardiovascular lineages suggest potentially significant roles for *Hox *genes in cardiovascular patterning and remodeling, nearly none of these have been defined *in vivo*. Perhaps one of the most confounding factors is an inherent difficulty in defining gene expression patterns in a structurally and physiologically diverse organ system branched throughout the entire body. To overcome this obstacle, we used *lacZ *reporter gene analysis in transgenic mice to obtain an approximate global view of the postnatal and adult vascular expression patterns of two *Hox *genes, *Hoxa3 *and *Hoxc11*. The data indicate distinct regionally restricted zones of expression that roughly correspond to the disparate embryonic expression domains of the two genes. These results support a role for *Hox *genes in specifying and maintaining positional identities of VSMCs and ECs in the adult vasculature.

## Materials and methods

### Cloning of *Hoxa3-lacZ*

A segment of genomic DNA of 11,473 base pairs (bp) located directly upstream of the *Hoxa3 *translational start codon was fused to *E.coli lacZ *derived from plasmid pCH110 (Amersham Pharmacia). The *Hoxa3 *fragment was derived from mouse genomic BAC clone RP24-353A14 (Children's Research Institute, Oakland, CA) and extended from the *NruI *restriction half-site located just 2 bp upstream of the *Hoxa3 *translational start codon [20] to the *BamHI *site at position -11,473 relative to the translational start. *Hoxa3-lacZ *was cloned in a pBluescript (Promega) -derived vector termed pSafyre [21] and released by *Spe1 *and *Not1 *restriction enzymes for the preparation of transgenic mice. Transgenic *lacZ *reporter studies showed previously that sequences located upstream of the *Hoxa3 *coding region contain both transcriptional promoter and enhancer functions capable of reconstructing the main aspects of endogenous *Hoxa3 *expression in E8.5-E9.5 mouse embryos (*Hoxa3 *reporter gene analysis: [22]; endogenous *Hoxa3 *expression: [22-24]).

### Transgenic mice

A *Hoxc11-lacZ *transgenic line carrying the LZc11-S construct was reported previously [25]. LZc11-S contained a 10 kb genomic fragment including the *Hoxc11 *transcription unit in addition to 2 kb and 5 kb of *Hoxc11 *upstream and downstream flanking sequences, respectively, as well as *E.coli lacZ *fused in frame to first exon coding sequences [25]; LZc11-S consistently reproduced endogenous *Hoxc11 *expression pattern in E11.5 – E13.5 embryos (n = 3 founders, including 2 transgenic lines). One of the LZc11-S lines has been designated TG(Hoxc11/lacZ)62D9Awg [26] and kept on FVB/NTac strain background. Mice from this line were used for *lacZ *expression analysis in blood vessels at postnatal and adult stages up to 1 year of age.

*Hoxa3-lacZ *mice were made according to standard transgenic procedures using single-cell FVB/NTac embryos. Transient transgenic mice (n = 6) and mice from one transgenic line designated TG(Hoxa3-lacZ)184H3Awg [26] were analyzed for reporter gene expression patterns at embryonic stage E14.5 (2 transient) and at 11 days *post natum *(p.n.) (2 transient), as well as at adult age of ≥ 6 weeks (line TG(Hoxa3-lacZ)184H3Awg, plus 2 transient transgenic mice). Patterns observed at E14.5 were consistent with the endogenous *Hoxa3 *expression patterns previously reported, albeit at earlier stages of embryonic development [22-24]. Among the postnatal and adult transient transgenic mice and the F1 mice of the TG(Hoxa3-lacZ)184H3Awg line, the vascular expression patterns observed at 11 days p.n. and at ≥ 6 weeks were consistent.

### X-Gal staining of *lacZ *reporter mouse tissue

Vascular tissues were fixed in a solution containing 0.2% glutaraldehyde, 2% paraformaldehyde, 2 mM MgCl_2 _in PBS at room temperature for periods ranging from 20 minutes to 1 hr depending on tissue. After rinsing for several hours in detergent solution containing 2 mM MCl_2_, 0.02% Nonidet P-40, 0.0001% Na-deoxycholate in PBS, the tissues were stained overnight at 32°C in a solution containing 20 mM Tris-HCl (pH 7.3), 2 mM MgCl_2_, 0.02% Nonidet P-40, 0.0001% Na-deoxycholate, 20 mM potassium-ferrocyanide, 20 mM potassium-ferricyanide, and 0.625 mg/ml X-Gal in PBS. After rinsing in PBS, stained tissues were fixed in 4% paraformaldehyde. For the preparation of frozen sections, stained tissues were infiltrated with 30% sucrose/PBS overnight at 4°C prior to embedding in OCT compound; tissues were cryosectioned and imaged using differential interference contrast (DIC) imaging.

### Reverse transcriptase (RT) – PCR

Under isoflurane anesthesia by inhalation, mouse tissues were prepared by transcardial perfusion with diluted (80%) RNAlater (Ambion, Austin, TX) to stabilize vascular tissue prior to excision followed by overnight incubation in undiluted RNAlater at 4°C. Vascular tissues were homogenized, and RNA was isolated with TRIzol Reagent (Invitrogen, Carlsbad, CA) according to manufacturers instructions and subsequently treated with RQ1 RNAse-free DNase (Promega, Madison WI). First-strand cDNA was prepared from total RNA using Superscript III First-Strand Synthesis System (Invitrogen, Carlsbad, CA). Gene-specific cDNA fragments were amplified with AmpliTaq Gold DNA Polymerase (Applied Biosystems, Foster City, CA) using the following primers: *Hoxa3 *(218 bp), F-GGGCACCGATGGCGTTGAGT and R-GCTGTGGTGGGGGCTGTGGA; *Hoxc11 *(311 bp), F-CCGGAGGAGGCAGGAGAAGA and R-CCGCCGCATAACAAGACGA. *Gapdh *primers were used for positive control reaction.

### Immunofluorescence (IF) labeling/Immunohistochemistry (IHC)

Under isoflurane anesthesia by inhalation, 8 week old normal FVB and TG(Hoxa3-lacZ)184H3Awg mouse tissues were prepared by transcardial perfusion with 0.1 M PIPES-buffered (pH 7.0) 2% paraformaldehyde followed by overnight immersion of excised tissues in PIPES-buffered 0.2% paraformaldehyde at 4°C. Carotid and dorsal aorta tissues were subsequently processed for cryosectioning (7 μm). For IF, rabbit anti-mouse primary antibodies for Hoxa3 (Santa Cruz Biotechnology, Santa Cruz, CA) and smooth muscle-specific Cy3-conjugated anti-Acta2 (Smooth muscle alpha actin [SMαA] Sigma, St. Louis, MO) were applied to carotid cross-sections at 10 μg/ml at room temperature for 2 hours following antigen retrieval with 10 mM Sodium Citrate (pH 6.0) buffer and brief microwave heating. For immunolocalization, fluorochrome-conjugated (Cy3 or Cy5) donkey anti-rabbit secondary antibodies (Jackson ImmunoResearch, West Grove, PA) were used. Cell nuclei were stained with Hoechst 33342. Fluorescence imaging at 400× was conducted using a Leica DMRB HC microscope, supported by a digital imaging workstation that includes a real time color digital camera (SPOT-RT) and a Gateway1400 PC.

For immunocytofluorescence (ICF), smooth muscle cells from dissociated vessel (see below) were immunolabeled with primary antibodies specific for either Hoxc11, β-gal, or Transgelin (SM22a; Abcam, Cambridge, MA) and detected with appropriate fluorochrome secondary antibodies followed by Hoechst 33342 nuclear labeling as described above. ICF/X-Gal double-labeling was accomplished by labeling the fixed cells with X-Gal prior to incubation with Cy3-conjugated anti-Acta2 antibodies and Hoechst 33342.

For IHC, rabbit anti-mouse primary antibodies for Hoxa3 were applied to dorsal aorta cross-sections at 10 μg/ml at room temperature for 2 hours following antigen retrieval with 10 mM Sodium Citrate (pH 6.0) buffer and brief microwave heating. Immunolocalization was detected with Vectastain ABC Kit utilizing peroxidase-conjugated anti-rabbit IgG antibodies and color detection with DAB, perioxidase substrate kit (Vector Labs, Burlingame, CA) according to manufacturers instructions. Micrographs of DAB-labeled sections were taken with differential interference contrast (DIC) imaging at 630×.

### Vessel explant cultures – *in vitro *assays

Small segments (2 mm length) of the lateral marginal vein and the femoral artery (mid-femoral region) of 6 weeks old *Hoxc11-lacZ *mice were dissected and placed into collagen-coated culture chambers. Explants were cultured in M199 medium supplemented with 20% FBS and pen-strep-glutamine. Outgrowing cells could be observed initially after ≈ 4 days, and cell density increased with prolonged culture.

To assess possible differential migration rates of transgene (*Hoxc11-lacZ*) expressing versus non-expressing cells, VSMCs from primary cultures were collected subsequent to the 5^th ^passage and plated in multi-well (4 wells) chambered slides at a concentration of approximately 500 cells/cm^2 ^in M199 media supplemented with 20% fetal calf serum (FCS). Cultures were allowed to grow to near confluence then "scratched" with a 200 μL pipette tip producing a 0.8 mm wound traversing the length of each chamber (2 cm). Under DIC imaging the wounded area was defined and marked for subsequent orientation at 100×. Cultures were allowed to grow for 24 hrs prior to processing for X-Gal labeling as described above with minor alterations: cells were fixed for 8 min prior to labeling. Using DIC imaging randomly selected fields were chosen from scratched areas of the four experimental wells and photographed at 100×; randomly selected fields from "normal" un-scratched areas were likewise imaged and photographed and used as negative controls. β-gal-positive (blue) and -negative cells of the experimental and control groups were scored and expressed as proportions of total number of cells and differences expressed as -fold change between groups. Data were expressed as mean ± standard deviation, and statistical significance (defined as *P *< 0.05) was determined using Student's *t*-test. For ICF experiments, cells were dissociated by enzymatic digestion (M199 media supplemented with 0.3 mg/ml elastase type III [Sigma, St. Louis MO], 1.8 mg/ml collagenase type I [Sigma, St. Louis MO] and 0.44 mg/ml soybean trypsin inhibitor [Sigma, St. Louis MO]) at 37°C for 1 hr prior to plating under the conditions as described above.

### Hoxc11 Antibody Production

Custom-made, affinity-purified, chicken-anti-mouse Hoxc11 antibodies were synthesized (Sigma Genosys, St. Louis MO) against an epitope of the Hoxc11 protein with the peptide sequence PPSTVTEILMKNEGS that comprises amino acid residues 106 – 120 of the variable region of the Hoxc11 protein; the human equivalent of this epitope was previously used successfully for raising HOXC11 antibodies [27]. Please note that the antibodies directed against this peptide are unable to recognize the Hoxc11-βgal fusion protein produced by the TG(Hoxc11/lacZ)62D9Awg mice as the *lacZ *coding region displaces Hoxc11 amino acids 96 – 304 in the transgene protein product [25]. Keyhole limpet hemocyanin (KLH) was selected as the peptide carrier protein and conjugation was achieved through attachment to the thiol-group of an additional cysteine residue placed at the N-terminus of the peptide sequence. The specificity of the affinity-purified antisera was determined by ELISA, as well as immunolabeling experiments with sagittal and cross sections of E12.5 mouse embryos that yielded detection patterns very similar to the familiar *Hoxc11 *RNA expression patterns ([28,29]; data not shown).

## Results

### Distinct *Hoxc11*- and *Hoxa3-lacZ *expression patterns in adult blood vessels

**Figure 1:**
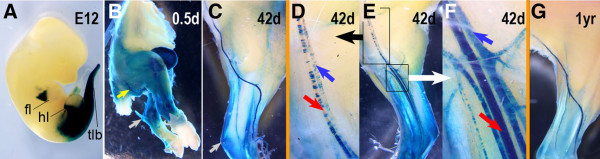
**Analysis of *Hoxc11-lacZ *reporter gene expression in hindlimb vasculature**. (A) E12 embryo of the transgenic shows posteriorly restricted *lacZ *reporter gene expression (blue) in most of the hindlimb region and tailbud, as well as in the posterior zeugopodal forelimb region. (B) X-Gal staining of posterior trunk and hindlimbs of 0.5 d newborn *Hoxc11-lacZ *transgenic mouse after skinning reveals expression in skeletal muscles and blood vessels (yellow arrow). (C) Lateral view of stained hindlimb of 42 d *Hoxc11-lacZ *transgenic mouse shows conspicuous β-gal activity in sciatic artery. (D-F) Medial view of same hindlimb as shown in panel C is presented in panel E and reveals distally restricted expression in both arteries and veins; close-up views of upper and lower sections of femoral artery (red arrows) and vein (blue arrows) are shown in panels D and F, respectively. (G) Lateral view of stained hindlimb from 1 yr old *Hoxc11-lacZ *transgenic mouse shows persistent reporter gene expression in blood vessels, most prominently in sciatic artery. All mice were derived from transgenic line TG(Hoxc11-lacZ)62D9Awg; fl: forelimb; hl: hindlimb; tlb: tailbud.

We previously reported that transgenic mice (n = 4 founders) carrying a *Hoxc11-lacZ *reporter gene construct in which *E.coli lacZ *with *SV40 *RNA processing signals was fused in-frame to *Hoxc11 *exon 1 coding sequences exhibited a conspicuous and reproducible β-gal expression pattern in mid-gestation embryos at ≈ E12 [25]. This pattern (Fig. 1A) was consistent with the posteriorly restricted pattern of endogenous *Hoxc11 *expression in par-axial and hindlimb mesenchyme as determined by ISH [25,28,29]. Accordingly, we concluded that this *Hoxc11-lacZ *construct included most of the control elements required for establishing the native *Hoxc11 *expression pattern in mid-gestation embryos [25,28,29]. Here, we used one of these *Hoxc11-lacZ *transgenic lines as a tool for gaining insight into global aspects of *Hoxc11 *expression during fetal and postnatal development, as well as in adulthood. This revealed β-gal activity in the hindlimb vasculature that had previously escaped our attention. Examination of young adult transgenic mice showed *lacZ *expression (X-Gal labeling) in all major blood vessels of the hindlimb, including arteries and veins (Fig. 1D–F) with strongest expression at the level of the zeugopod and lower stylopod (femur). In several vessels, including the femoral artery, X-Gal labeling was rather uniform in the lower limb and became progressively mosaic in upper femoral limb regions near the expression boundary (Fig. 1E, F). However, vessels showing mosaic β-gal expression patterns could also be seen next to vessels that appeared to be uniformly stained at a given proximal-distal level of the zeugopod. Persistent vascular expression was observed in animals of 1 year of age (Fig. 1G), the latest stage examined.

In addition to expression in blood vessels, we observed strong and specific *Hoxc11-lacZ *activity in kidney, ureter, urinary bladder, and uterus (data not shown). This activity in both the male and female urogenital system is consistent with previously reported endogenous *Hoxc11 *expression in the metanephric mesenchyme during mid-gestation, and in cortical regions of the developing kidney during later development [28], as well as *HOXC11 *expression in human uterine endometrium [30]. Furthermore, we also observed diffuse β-gal activity in hindlimb cartilage and skeletal muscle of postnatal and adult mice (Fig. 1B, C, E); although this activity apparently became gradually weaker and increasingly restricted to distal limb regions with age, it was still clearly detectable in 1 year old mice (Fig. 1G). This may indicate that endogenous *Hoxc11 *expression in skeletal muscle, first observed in embryonic myotomes and developing muscle during fetal development [28], persists in adulthood.

**Figure 2:**
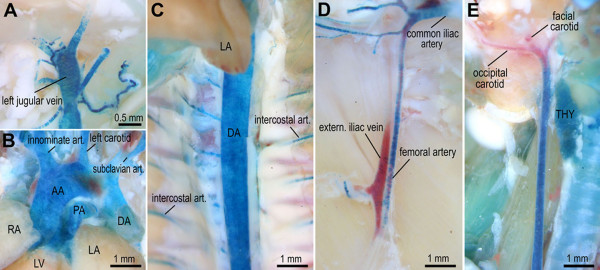
***Hoxa3-lacZ *expression in major blood vessels**. (A-E) Prominent expression (blue) was detected by whole-mount X-Gal staining of 6 wk old *Hoxa3-lacZ *transgenic mice in external jugular vein (panel A), aortic arch (AA) and all major vessels in the aortic arch region including innominate and left carotid artery, pulmonary vein (PA), and subclavian artery (panel B); strong expression was also detected in the descending thoracic aorta (DA) and intercostal arteries (panel C), as well as the common iliac artery and proximal femoral artery (panel D); *Hoxa3-lacZ *expression in the common carotid artery exhibits a distinct anterior boundary near the branch point into facial and occipital carotid arteries (panel E); anterior points to the top in panels A-C, and E, and to the right in panel D; please note the distal expression boundary in the femoral artery just below the branch point of the internal iliac. LA: left atrium; LV: left ventricle; RA: right atrium; thy: thyroid gland.

To gain further support for the emerging concept of distinct zones of *Hox *activity in the adult vascular network, we prepared a *Hoxa3-lacZ *transgene construct that contained 11.5 kb of genomic sequences located directly upstream of the *Hoxa3 *translational start site (see Materials and Methods). Analysis of independent *Hoxa3-lacZ *transgenic mice (n = 4) at 11 d, 6 wks, and ≥ 8 wks *post natum *revealed a reproducible pattern of *lacZ *expression in most major blood vessels (common carotid arteries, aortic arch, dorsal aorta and inter-costal arteries, pulmonary and renal arteries, cardinal and jugular veins, proximal femoral arteries and veins) (Fig. 2A–E). Remarkably, the distinct anterior expression boundary near the cranial branch point of the common carotid arteries (Fig. 2E) was approximately at the same anterior-posterior level as the expression boundary in the jugular veins (Fig. 2A), and these expression boundaries roughly correspond to the anterior *Hoxa3 *expression limit at the somite s4/5 boundary during embryonic development [22]. *Hoxa3-lacZ *expression in the dorsal aorta and inferior vena cava extended beyond the iliac branching point into the most caudal blood vessels of the tail, as well as the proximal limb vessels, including expression in the proximal segments of the femoral artery and vein that reached just to the branch of the epigastric artery (Fig. 2D).

**Figure 3:**
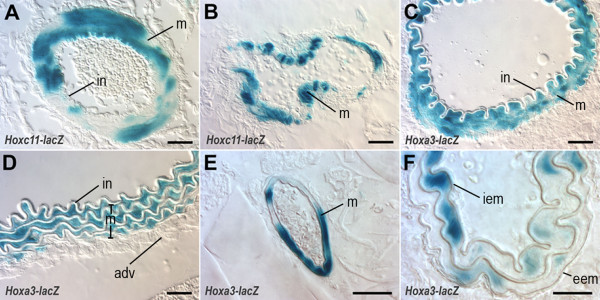
***Hoxc11- *and *Hoxa3-lacZ *expression patterns in blood vessel sections of 6 wk old transgenic mice**. (A, B) Cross sections of the distal femoral artery (panel A) and femoral vein (B) show restriction of *Hoxc11-lacZ *expression in subsets of VSMCs in arterial and venous vessel walls as indicated by X-Gal staining (blue). (C-F) Cross sections of X-Gal-stained iliac artery (C), descending thoracic aorta (D), intercostal artery (E), and common carotid artery (F) of *Hoxa3-lacZ *transgenic mice indicate strong β-gal expression (blue) in the VSMCs of the vessel walls, whereas X-Gal staining in the flattened endothelial cells lining the intimal laminae is difficult to discern. adv: adventitia eem: external elastic membrane, iem: internal elastic membrane, in: intima, m: media. Space bars: 25 μm in all panels.

To determine reporter gene expression patterns in vessel walls, we analyzed sections of X-Gal-stained blood vessels from adult *Hoxc11-lacZ *and *Hoxa3-lacZ *mice (≥ 6 wks). As exemplified by data shown in Figure 3, *Hoxc11-lacZ *expression was observed predominantly, if not exclusively, in subsets of VSMCs of the major blood vessels of the lower hindlimb, including arteries (Fig. 3A) and veins (Fig. 3B). Overall, the *Hoxa3-lacZ*-expressing vessel walls of the iliac artery, the descending thoracic aorta, the intercostal arteries, and the common carotid arteries (Fig. 3C–F) exhibited similar patterns as the *Hoxc11-lacZ*-expressing walls of the lower limb, including prominent expression in VSMCs. Due to their flat morphology, expression in epithelial cells lining the intimal lamina was difficult to discern based on X-Gal staining in either case (i.e. *Hoxc11-lacZ *and *Hoxa3-lacZ *transgenic mice). This was resolved by performing immunofluorescent labeling studies using β-gal-specific antibodies, which detected endothelial β-gal expression in the common carotids of *Hoxa3-lacZ *mice, but not in the tibial/femoral arteries of *Hoxc11-lacZ *mice (data not shown).

### Validation of *Hoxc11 *and *Hoxa3 *reporter gene expression patterns

**Figure 4:**
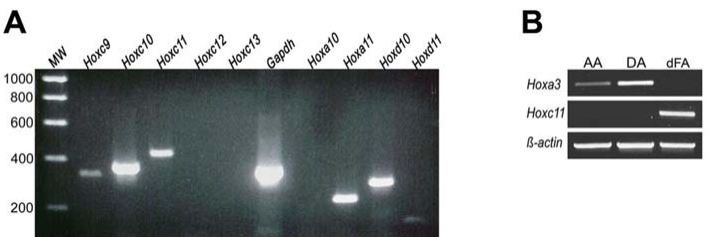
**RT-PCR analysis of *Hox *gene expression in adult blood vessels**. (A) Total RNA derived from hindlimb blood vessels (6 wk old FVB/NTac mice) as defined in the text was used for cDNA synthesis, and *Hox-*specific cDNA fragments were amplified by using primers specific for the *AbdB*-type *Hoxc *genes; this resulted in PCR products in the expected size range for *Hoxc9 *(323 bp), *Hoxc10 *(350 bp), and *Hoxc11 *(435 bp); PCR reactions with primers specific for the *Hoxc11 *and *Hoxc10 *paralogous genes (*Hoxa11 *and *Hoxd11*, as well as *Hoxa10 *and *Hoxd10*) resulted in amplification products in the expected size range for *Hoxa11 *(220 bp), *Hoxd11 *(131 bp), and *Hoxd10 *(286 bp), whereas no product was detected for *Hoxa10; *positive control reaction: *Gapdh*-specific primers; MW: molecular weight standards in base pairs (bp). (B) RT-PCR analysis of *Hoxa3 *and *Hoxc11 *expression in adult mouse (6 wk) blood vessel segments including aortic arch (AA), descending thoracic aorta (DA), and distal femoral artery (dFA); positive control reaction was performed with *β-actin*-specific primers.

To determine whether the vascular *Hoxc11- *and *Hoxa3-lacZ *reporter gene expression reflected corresponding endogenous gene activities, we performed semi-quantitative reverse transcriptase (RT)-PCR analysis of total RNA isolated from defined vessel segments of 6 wk old mice. For a first set of reactions, we used RNA isolated from femoral artery and vein segments ranging from the mid-femoral region just proximal to the *Hoxc11-lacZ *expression boundary to the distal femoral artery branch point approximately at knee-level. In addition to examining this RNA sample for the presence of *Hoxc11*-specific transcripts, we determined whether there was evidence for expression of the remaining *AbdB*-type *Hoxc *genes (*Hoxc9, Hoxc10, Hoxc12*, and *Hoxc13*), as well as for the *Hoxc11 *and *Hoxc10 *paralogous genes (*Hoxa11, Hoxd11*, and *Hoxa10, Hoxd10*). The results indicate strong expression of *Hoxc11 *and *Hoxc10*, whereas *Hoxc9 *expression appeared less abundant, and expression of *Hoxc12 *and *Hoxc13 *was not detectable (Fig. 4A). Strong expression was also indicated for two of the paralogous genes, *Hoxa11 *and *Hoxd10*, whereas no RT-PCR amplification product was detected for *Hoxa10*, and the band corresponding to *Hoxd11 *was weak (Fig. 4A). These data suggest selective activity of *AbdB-*type *Hoxc *genes in major blood vessels of the mid-femoral hindlimb region. Specifically, the detection of *Hoxc11*-specific amplification products suggests that the vascular *Hoxc11-lacZ *reporter gene expression observed in this region reflects authentic *Hoxc11 *transcriptional activity. The detection of *Hoxa11- *and *Hoxc9-specific *amplification products is consistent with the isolation of *HOXA11 *and *HOXC9 *cDNAs from human smooth muscle cell cDNA libraries reported previously [18].

To obtain supporting evidence for regionally restricted expression of endogenous *Hoxa3 *and *Hoxc11 *within the vascular network, we isolated RNA from the aortic arch, the descending thoracic aorta, and the distal femoral artery (the distal femoral artery segment used was as defined above) for RT-PCR analysis. In agreement with the *Hoxa3- *and *Hoxc11-lacZ *reporter gene analysis, the data indicate *Hoxa3 *expression in the aortic arch and the descending thoracic aorta but not in the distal femoral artery, whereas *Hoxc11 *expression is detectable exclusively in the latter (Fig. 4B).

**Figure 5:**
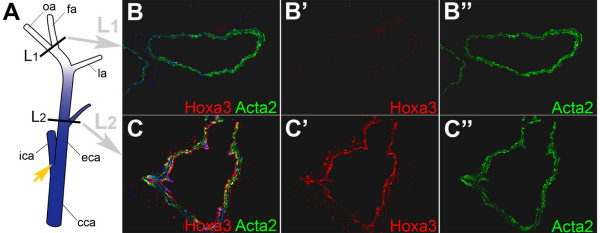
**Validation of endogenous *Hoxa3 *expression boundary in adult carotid artery of FVB/NTac mouse**. (A) Diagram of right carotid segment indicating reporter gene expression domain (blue shading) with anterior boundary at level of lingual artery (la) as observed in *Hoxa3-lacZ *transgenic mouse (see Fig. 2E for comparison); yellow arrow points to carotid bifurcation into internal (ica) and external carotid arteries (eca), respectively; sections corresponding to levels L1 and L2 as indicated in the cartoon were used for detection of Hoxa3 and Acta2 by immunofluorescence as shown in the three upper (B,B'B") and lower (C,C',C") panels to the right. Both L1 and L2 sections were incubated with Hoxa3-specific and Cy3-conjugated Acta2-specific antibodies; using Cy5-conjugated secondary antibodies, red-fluoresent-labeled Hoxa3 protein was detected only in section L2 (panel C'), whereas green-fluorescent-labeled Acta2 expressed in VSMCs was detected in both L1 and L2 sections (panels B" and C"); accordingly, multichannel composite micrographs (panels B and C) for detecting red (Cy5), green (Cy3) and blue (Hoechst 33342 for nuclear labeling; this is not shown individually) indicate co-localization of Hoxa3 and Acta2 (yellow) in VSMCs only in section L2 (panel C). fa: facial artery; oa: occipital artery.

**Figure 6:**
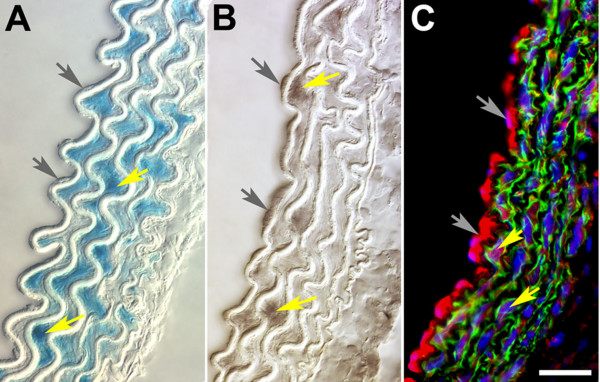
**Comparison of *Hoxa3-lacZ *reporter and endogenous Hoxa3 protein expression in VSMCs and ECs of aortic arch**. (A) Cross section of aortic arch from adult *Hoxa3-lacZ *mouse after X-Gal-staining indicates reporter gene expression (blue) in VSMCs (yellow arrows) whereas expression in cells of the endothelial layer (gray arrows) is difficult to discern. (B) Immunohistochemical detection of Hoxa3 in comparable section of adult (8 wk) dorsal aorta; immunolabeling of section with anti-Hoxa3 antibodies and subsequent colorimetric detection (DAB-Ni) reveals expression in ECs (gray arrows) and vascular VSMC (yellow arrows). (C) Multichannel composite micrograph of adult aortic arch section after incubation with immunoreagents specific for Hoxa3 (Cy5-red) and Acta2 (Cy3- green), and Hoechst 33342 for nuclear labeling. The bright red labeling in ECs (gray arrows) confirms Hoxa3 expression. The purple labeling in the media indicates Hoxa3 expression in certain nuclei of VSMCs (yellow arrows). Green label indicates that Acta2 expression is restricted to the media. Lumen is to the left in all panels; space bar: 25 μm.

Evidence that the anterior *Hoxa3-lacZ *boundary in carotid arteries (Fig. 2E) reflects the endogenous *Hoxa3 *pattern in these vessels was provided at the protein level by immunolabeling studies with Hoxa3-specific antibodies that detected Hoxa3 protein exclusively in sections of carotid vessel segments located posterior of this boundary (Fig. 5). Furthermore, while *Hoxa3-lacZ *expression in the endothelial layer was difficult to discern by X-Gal staining as exemplified in Figure 6A, endogenous Hoxa3 protein expression in both ECs and VSMCs of the aortic arch was confirmed by immunolabeling (Fig. 6B,C). Combined, these data strongly suggest that *Hoxa3-lacZ *mimics the authentic *Hoxa3 *expression pattern in adult blood vessels. The *Hoxa3-lacZ *patterns correlate remarkably well with the broad spectrum of vascular defects previously observed in *Hoxa3 *null mice, which included abnormalities of the carotid system, thinning of the aorta and enlargement of major veins [4,6]. Lack of information about the *Hoxa3 *vascular expression pattern, however, largely precluded interpretation of these data with regard to potential underlying mechanisms.

### *Hoxc11-lacZ *expression *ex vivo*

**Figure 7:**
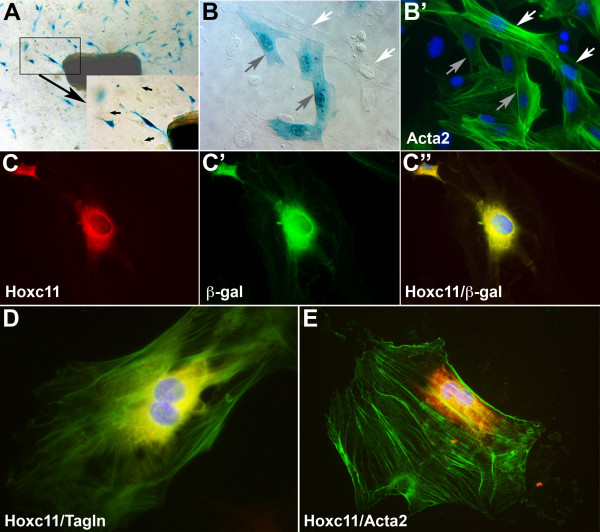
***Hoxc11-lacZ *expression is restricted to a subset of smooth muscle cells in vessel explant culture**. (A) Cellular outgrowth of explanted femoral artery/lateral marginal vein vessel segments (~2 mm; central dark ovoid structure in panel A) from young adult *Hoxc11-lacZ *transgenic mice [TG(Hoxc11/lacZ)62D9Awg] is comprised of β-gal-positive (blue) and -negative cells (black arrows in panel A, inset). (B) X-Gal labeling of cultured, femoral artery SMCs of *Hoxc11-lacZ *reporter mice reveals β-gal-positive (blue) and β-gal-negative cells as indicated by grey and white arrows, respectively. (B') Subsequent to X-Gal labeling, the cells shown in panel B were immunolabeled with antibodies specific for SMC marker Acta2 (formerly known as SMαA) as indicated by green fluorescence; note that both β-gal-positive and -negative cells as marked by grey and white arrows, respectively (grey and white arrows correspond to the same cells in panels B and B'), are labeled by anti-Acta2; nuclei were labeled with Hoechst 33342 (blue). (C/C'/C") Double-labeling of explant-derived, cultured femoral artery SMCs of TG(Hoxc11/lacZ)62D9Awg mice with antibodies against Hoxc11 (red signal, panel C) and anti-β-gal (green signal, panel C') results in a merged yellow signal in panel C". (D/E) Double-immunolabeling of cultured mitotic femoral artery SMCs with antibodies against Hoxc11 (red signal) and two standard SMC markers, including Transgelin (Tagln, formerly known as Smooth muscle protein 22-alpha; green signal, panel D), and Acta2 (green signal, panel E); note, overlap between Hoxc11 and Tagln signals in panel D results in yellow fluorescence, whereas the lesser degree of overlap between Hoxc11 and Acta2 signals in panel E results in discernible Hoxc11 (red) signal in the peri-nuclear region. Nuclei (blue) were labeled with Hoechst 33342.

Blood vessel explants (segments of ≈ 2 mm length) of the lateral marginal vein and the distal femoral artery of 6 wk old *Hoxc11-lacZ *mice were cultured to determine whether reporter gene activity was retained in outgrowing cells. Initial outgrowth was observed after ≈ 4 days under standard conditions (M199 medium supplemented with 20% FBS and pen-strep glutamine; see Materials and Methods), and X-Gal staining after 11 days of culture consistently showed β-gal activity in a fraction of the outgrowing cells (Fig. 7A, B), while control explants from corresponding regions of non-transgenic mice did not produce β-gal-positive cells (not shown). Co-labeling with antibodies directed against a common marker for smooth muscle cells (SMCs), i.e. Acta2 (previously known as SMαA) showed that the β-gal-positive cells expressed Acta2, whereas not all of the Acta2-expressing cells were β-gal-positive (compare panels B and B' in Fig. 7). Using Hoxc11- and β-gal-specific antibodies (see Materials and Methods for preparation and testing of Hoxc11-specific antibodies) in double-immunolabeling studies, we determined that these β-gal-positive cells do express endogenous Hoxc11 (Fig. 7C,C',C"). Furthermore, using the Hoxc11 antibodies in double-immunolabeling assays with Acta2 antibodies, as well as antibodies against a second SMC marker, Transgelin (Tagln; previously known as SM22) showed co-labeling in the perinuclear region of individual cultured VSMCs from the femoral artery of TG(Hoxc11/lacZ)62D9Awg mice in both cases (Fig. 7D,E).

**Figure 8:**
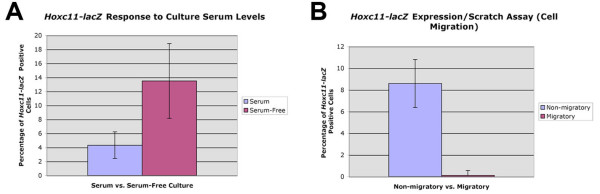
**Functional assays of *Hoxc11-lacZ *expression in cell culture**. (A/B) Explant cultures. (A) The proportion of β-gal-positve cells derived from explant cultures of lateral marginal veins and femoral arteries from 6 weeks old *Hoxc11-lacZ *mice is approximately 4.3% (± 1.92%) under culture conditions that include 20% fetal bovine serum; this increases to approximately 3-fold to 13.5% (± 5.34%) under serum-free conditions; *P *< 0.001. (B) Cell migration/scratch assay indicates a dramatic (71-fold) reduction in transgene expression in the group of migratory cells found in the scratch area, (8.6% ± 2.2 blue cells in normal (unwounded) area compared to 0.11% ± 0.46 in wounded area); difference of approximately 71-fold, *P *< 0.001.

Continued *Hoxc11-lacZ *expression in only a subpopulation of cultured VSMCs raised the question whether these cells might be functionally distinct compared to β-gal-negative cells. This was addressed in a preliminary manner by performing two simple and straightforward *in vitro *assays that tested response to culture serum levels and migration behavior. The results show that the proportion of *Hoxc11-lacZ*-expressing cells increased approximately 3-fold to 13.5% under serum-free culture conditions compared to about 4.3% under conditions including 20% FBS (Fig. 8A). In scratch assays, the proportion of β-gal-positive cells was dramatically reduced (71-fold) among the migratory cells found in the scratch area compared to the cells residing in the undisturbed monolayer (Fig. 8B). These differential reporter gene expression patterns in cultured VSMCs undergoing phenotypic modulation suggest that *Hoxc11 *expression is associated with distinct VSMC phenotypes.

VSMCs are capable of exhibiting a wide range of different phenotypes in response to changes in local environmental cues, a phenomenon known as phenotypic modulation or phenotype switching [31,32]. The morphologically and functionally distinct synthetic and contractile VSMCs are considered to mark the boundaries at the opposite ends of a spectrum within which VSMCs can modulate their phenotypic properties [33]. The decreased *Hoxc11-lacZ *expression in migrating cells is consistent with the lower level of reporter gene activity in response to serum – in both cases the phenotypic equilibrium is shifted towards a proliferative and synthetic phenotype [32]. Accordingly, these data suggest preferential *Hoxc11 *activity in contractile VSMCs of spindle-like morphology.

## Discussion

**Figure 9:**
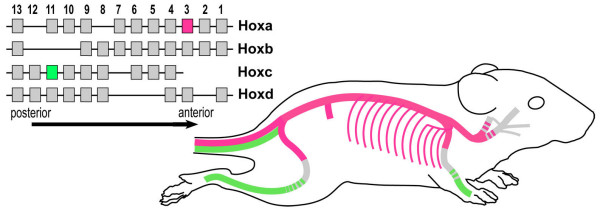
**Summary of *Hoxa3 *and *Hoxc11 *expression domains in major blood vessels as indicated by reporter gene analysis**. Left, diagram of the four *Hox *clusters shown in parallel alignment with arrow at bottom indicating transcriptional orientation and spatial co-linearity of *Hox *map positions with distinct expression domains along the longitudinal body axis during embryonic development. Right, cartoon of mouse depicting *Hoxa3-lacZ *(red) and *Hoxc11-lacZ *(green) zones of expression in major blood vessels.

During development, a primary role of *Hox *genes is thought to "translate" positional information established within a spatial coordinate system into positional identities, which is essential for the differentiation of defined body structures at their appropriate anatomic sites [34]. Once adult structures and organs have formed, a fundamental question arises regarding how their organo-typic shape and physiological properties are being maintained. According to a recently proposed concept this may be achieved by retaining positional identities specified by a *Hox *code [35,36], which is initially set up during embryonic patterning [37]. This positional identity model was supported by gene expression profiling of human fibroblast populations derived from distinct adult anatomic sites, which revealed that the site-specific *Hox *profiles mirrored the embryonic *Hox *patterns relative to the main developmental axes. This was interpreted as evidence for a *Hox *code-based positional memory underlying the control of topographic fibroblast differentiation [35,36]. The results presented here show complex, yet distinct, spatially restricted *Hoxa3 *and *Hoxc11 *patterns in adult blood vessels (see Fig. 9 for a schematic summary) that largely correspond to the embryonic *Hoxa3 *[23,24] and *Hoxc11 *[25,28] activity domains. These data are consistent with the positional identity model and support the idea of discrete *Hox-*specified identities in the vascular network. Validation of this concept in future studies will require a comprehensive analysis of the spatial expression patterns of all 39 *Hox *genes in the developing and adult cardiovascular system.

In keeping with the positional identity model, recent global gene expression profiling revealed that similar to fibroblasts, ECs and SMCs exhibit considerable diversity depending on function and anatomic site [38,39]. Within the cardiovascular system, this is likely to reflect diverse physiological requirements, including regional variations in vessel tone, blood flow, blood pressure, oxygen content, nutrient content, etc., and *Hox *genes are considered excellent candidates for orchestrating the corresponding regional gene expression profiles [3,38,39]. Data demonstrating that genes encoding SMC-restricted proteins are direct targets of Hox transcription factors [40] are consistent with this model. Furthermore, several *Hox *genes (including *Hoxa3*) have been implicated in controlling the conversion of ECs to the angiogenic phenotype [41], which is of great relevance for tumor-angiogenesis, as well as for neo-vascularization during wound healing and other normal adult physiological activities. Likewise, phenotype switching of VSMCs is known to be associated with a wide range of diseases of the cardiovascular system including atherosclerosis, stroke, high blood pressure, aneurisms, etc. [32]. Our data showing *Hoxc11 *expression to be restricted to a subset of VSMCs derived from the same vessel and anatomic site as VSMCs not expressing *Hoxc11 *may indicate that *Hox *expression profiles are associated with distinct VSMC phenotypes. This proposition is further underscored by the preliminary results from our functional assays suggesting a role for *Hoxc11 *in defining phenotypic properties of VSMCs. Taken together, elucidating the role of *Hox *genes in adult blood vessels is likely to be critically important for understanding vascular disease mechanisms.

## Conclusion

Our results support a conceptual model of *Hox-*specified positional identities in adult blood vessels, which is of likely relevance for understanding the mechanisms underlying regional physiological diversities in the cardiovascular system. The data also demonstrate that conventional *Hox *reporter gene mice are useful tools for visualizing complex *Hox *expression patterns in the vascular network that might be unattainable otherwise. Importantly, these mice are a valuable resource for the isolation and phenotypic characterization of specific subpopulations of vascular cells marked by distinct *Hox *expression profiles.

## Authors' contributions

NDP performed the reporter gene and immunolabeling studies, RT-PCR analysis, and functional assays with cultured cells and contributed to the data analysis and the preparation of the manuscript. RPV participated in the design and interpretation of the immunolabeling experiments and the interpretation of pertinent results. DFJ made the *Hoxa3-lacZ *transgenic mice and helped with finalizing the manuscript. DM contributed to the histological analysis of X-Gal-stained blood vessels and participated in preparing the manuscript. TM participated in planning the experiments and discussing the results. JPS helped with interpreting the lacZ staining and contributed to the final version of the manuscript. AA designed this study, cloned the *Hoxa3 *reporter gene construct, and drafted the original manuscript. All authors have read and approved the manuscript.
